# Margret M. and Paul B. Baltes Award

**DOI:** 10.1093/geroni/igaa057.3201

**Published:** 2020-12-16

**Authors:** Merril Silverstein

## Abstract

The lecture will be given by the2019 Baltes Award recipient, Allison Bielak, PhD, FGSA, of Colorado State University. The recipient of the2020 Baltes Award is William J. Chopik, PhD. The Margret M. and Paul B. Baltes Foundation Award in Behavioral and Social Gerontology recognizes outstanding early-career contributions in behavioral and social gerontology. The award is generously funded by the Margret M. and Paul B. Baltes Foundation.

